# A Cross-Sectional Study of Occupational Noise Exposure and Hypertension in Malaysia

**DOI:** 10.7759/cureus.48758

**Published:** 2023-11-13

**Authors:** Felicia Yan Lin Lee, Nor Halizam Ismail, Pui Mun Liew, Sin How Lim

**Affiliations:** 1 Social and Preventive Medicine, University Malaya, Kuala Lumpur, MYS; 2 Health and Environment, Kuala Lumpur City Hall, Kuala Lumpur, MYS; 3 Ang Mo Kio Polyclinic, National Healthcare Group Polyclinics, Kuala Lumpur, MYS

**Keywords:** non-auditory health effects, non-communicable disease, noise hazard, hypertension, occupational noise exposure

## Abstract

Introduction

Noise is an occupational hazard that has been linked to negative effects beyond the auditory system, including hypertension. This study investigated the associations between occupational noise exposure and the risk of hypertension.

Methods

This cross-sectional study was conducted among state government employees. Data were collected through an online, self-administered questionnaire. Participants were divided into noise-exposed and non-noise-exposed groups based on their self-reported speech communication effort. Hypertension status was also self-reported. Descriptive analysis was performed on sociodemographic, lifestyle, medical history, and occupational characteristics to determine frequency and prevalence. Multiple logistic regression was conducted to compute the odds ratio (OR) and 95% confidence interval (CI) while adjusting for potential confounders.

Results

A total of 1005 state government employees were analyzed. The prevalence of hypertension was 18.8% among noise-exposed employees. After adjusting for age, sex, diabetes, dyslipidemia, BMI, and family history of hypertension, diabetes, and dyslipidemia, noise-exposed employees had a higher risk of hypertension, with an OR of 1.70 (95% CI = 1.09-2.66, p = 0.020), compared to non-exposed employees. Noise-exposed employees who had been exposed to occupational noise hazards for more than 10 years had a higher risk of hypertension (OR 2.04, 95% CI = 1.26-3.29, p = 0.004) compared to those who were unexposed.

Conclusion

Occupational noise exposure was associated with an increased risk of hypertension. These findings underscore the need to address noise exposure in the workplace and implement appropriate strategies to reduce its potential negative impact on employees' health.

## Introduction

Hypertension is the most comprehensively studied risk factor for cardiovascular disease (CVD). The global disease burden has shifted from communicable to non-communicable diseases (NCDs) in recent years [1]. This shift has led to increased interest in the effects of occupational factors, such as noise exposure, on the development of CVD risk factors (i.e., hypertension). A report from the United States (U.S.) indicated that 14% of hypertensive workers had a history of noise exposure [2].

Globally, 22% of major occupational risks are attributed to noise hazards, making it the second most significant occupational burden of disease globally, after physical injuries [3]. Hearing impairment is a well-known effect of occupational noise, as it can directly injure the auditory system [4,5]. However, the non-auditory effects of noise, such as stress, cognitive impairment, and CVD, including hypertension, ischemic heart disease, and stroke, can be just as harmful as effects on hearing [6,7]. Based on Babisch's noise effect model, noise hazards have been linked to negative effects beyond the auditory system as a result of a stress cascade [8]. The general theory posits that noise acts as a nonspecific biological stressor, affecting the endocrine and autonomic nervous systems and leading to changes in body homeostasis. According to Golmohammadi et al. (2022) [9], psychophysiological responses such as stress and cognitive impairment may also begin to manifest at noise levels above 65 dB(A) and 55 dB(A), respectively. Prolonged noise exposure can lead to changes in the body's systems, potentially affecting blood pressure regulation, lipid metabolism, and glucose metabolism [8]. Six systematic reviews on the association between occupational noise exposure and hypertension have been published over the past decade, with a combined risk estimate ranging from 1.08 to 2.56 [10].

Noise, defined as "any unwanted or undesirable sound," is a pervasive but often overlooked aspect of our daily lives [11]. In the workplace, daily activities engaged in by workers can pose various hazards to their safety and health, including noise. It is estimated that around 22 million workers in the U.S. are exposed to this occupational hazard each year [12]. Additionally, an average of 28% of European workers reported a history of noise exposure for at least a quarter of their working hours [13]. High levels of noise exposure are closely associated with certain work tasks, particularly those involving impact processes, heavy machinery, or generators. Industries with a high risk of noise exposure include construction, manufacturing, mining, repair work, and transportation [2,14].

There have been several studies on the effects of noise exposure on hypertension in certain industries, such as aircraft, manufacturing, railway, and road traffic [2,14]. However, much less research has been conducted on these effects among the landscaping and pest control services industries. In these sectors, workers are often exposed to noise during their work, both internationally and in this region. Despite the substantial number of workers exposed to occupational noise in the ASEAN region, including Malaysia, there has been a lack of research on the effects of this exposure on hypertension in this area, making the research particularly relevant and important. By filling this gap in the literature, this study aims to shed light on an important and understudied topic that may have significant implications for the health and well-being of workers in this region.

The objective of this paper was to determine the association between occupational noise exposure and hypertension in the Malaysian setting.

## Materials and methods

Subjects

In this cross-sectional study, universal sampling was used to recruit employees from a state government in Malaysia between April and October 2022. Participants were divided into noise-exposed and non-noise-exposed groups based on their self-reported speech communication effort. This classification used a question adapted from the Checklist for Identification of Excessive Noise in the Industry Code of Practice (ICOP) for Management of Occupational Noise Exposure and Hearing Conservation 2019, developed by the Department of Occupational Safety and Health (DOSH), Ministry of Human Resources, Malaysia. The noise-exposed group is defined as "employees who are assigned to a work area with unwanted sounds (loud enough requiring one to speak in a raised voice to be heard at one meter away) throughout the work week" [15]. Participants were excluded if the duration of noise exposure was less than five years for noise-exposed employees, if they had a history of congenital hearing impairment, history of head trauma requiring hospitalization, were pregnant, or had previous occupational noise exposure and shift work. Data were collected through an online, self-administered questionnaire distributed in both English and Bahasa Malaysia. The questionnaire included questions on demographic characteristics, smoking status, medical history, recreational noise exposure, environmental noise exposure, stress, sleep disturbance, and personal hearing protection (PHP) usage.

Recreational noise exposure is defined as "any exposure to headphones/earphones, live music, nightlife, sports-related noise, or cinema once a month and above for the past 12 months," based on an adapted questionnaire by Armitage et al. [16]. Environmental noise exposure is defined as "place of residence located in an area with loud noise; for example, near a highway, factory, or airfield" [15]. Stress measurement was collected using the stress domain of the Depression Anxiety Stress Scale (DASS-21) questionnaire. It consists of a total of seven self-reported items, comprising Questions 1, 6, 8, 11, 12, 14, and 18. It was also validated in the Malay language with Cronbach's alpha values of 0.79 for stress. Its factor loading values for each item range from 0.20 to 0.72. Stress is defined as "DASS-21 Questionnaire Stress Domain Score >7" [17]. Sleep quality measurement was collected using the Epworth Sleepiness Scale (EES). It consists of a total of six self-reported items and had a high level of internal consistency both in physical and online formats, with Cronbach's alpha values of 0.88 and 0.85, respectively. It was also validated in the Malay language. Sleep disturbance is defined as "ESS Score >10" [18]. Personal hearing protection (PHP) usage is defined as "wearing PHP throughout the work process" [15].

To enhance the accuracy of statistical analysis, individuals who declined to participate, provided incomplete information, or did not respond were excluded from the current research.

Hypertension

It is based on the participants' report of ever receiving treatment for hypertension, having been informed by a medical doctor or other healthcare professional that they have hypertension.

Statistical analysis

Data were analyzed using the IBM SPSS Statistics for Windows, Version 28 (Released 2021; IBM Corp., Armonk, New York). Descriptive analysis was used to describe the sociodemographic, occupational, and medical characteristics of the participants. The chi-square (χ^2^) test was used to determine the association between occupational noise exposure and hypertension among state government employees. Independent variables with a p-value less than 0.25 were selected to proceed with multivariable logistic regression to determine the association between occupational noise exposure and hypertension, as well as its covariates (age, sex, family history, diabetes, dyslipidemia, smoking, BMI, alcohol consumption, stress, sleep disturbance, environmental noise exposure, and recreational noise exposure). Further analysis was also conducted, wherein noise exposure status was categorized according to the duration of noise exposure and PHP usage among noise-exposed employees. Adjusted odds ratios (aOR) and 95% confidence intervals were used to determine the strength of associations between the variables. Statistical significance was set at a p-value less than 0.05.

Ethical considerations

Ethical approval for this study was granted and registered with the National Medical Research and Ethics Committee (NMRR ID-22-00859-JWI). Participants' privacy and confidentiality were maintained and protected, as no personal identifiers were collected in this study.

## Results

Characteristics of subjects

The final number of the study group comprised 1005 employees with a total number of 1013 employees being excluded. The basic characteristics of the subjects are shown in Table 1. In this study, 66.9% of the participants were under 40 years old, and 73.8% were male. With respect to education level, 68.9% had attended at least secondary school education, while 22.8% had a tertiary education. And 36.3% were current smokers. Among the 1005 employees, 49.9% were noise-exposed employees. The duration of employment for this sample population was primarily skewed toward those with more than 10 years of employment, with 79.0% falling into this category and only 21.0% having been employed for less than 10 years. Among the noise-exposed employees, most of them (56.3%) have been exposed to noise for more than 10 years and 67.3% wore PHP throughout their work process.

**Table 1:** Characteristics of employees (N = 1005)

Variables	N (%)	Noise-exposed employees, n (%)	Non-noise-exposed employees, n (%)	p-value
	(n = 1005)	(n = 501)	(n = 504)	
Age (years)				0.001*
<40	672 (66.9)	359 (71.7)	313 (62.1)	
≥40	333 (33.1)	142 (28.3)	191 (37.9)	
Sex				<0.001*
Male	742 (73.8)	472 (94.2)	270 (53.6)	
Female	263 (26.2)	29 (5.8)	234 (46.4)	
Family history of hypertension/dyslipidemia/diabetes mellitus				0.788
Yes	636 (63.3)	315 (62.9)	321 (63.7)	
No	369 (36.7)	186 (37.1)	183 (36.3)	
BMI				0.343
Underweight	19 (1.9)	6 (1.2)	13 (2.6)	
Normal	305 (30.3)	195 (38.9)	194 (38.5)	
Overweight	389 (38.7)	152 (30.3)	140 (27.8)	
Obese	292 (29.1)	148 (29.5)	157 (31.2)	
Smoking status				<0.001*
Current smoker	365 (36.3)	257 (51.3)	108 (21.4)	
Ex-smoker	79 (7.9)	52 (10.4)	27 (5.4)	
Never smoked	561 (55.8)	192 (38.3)	369 (73.2)	
Diabetes mellitus				0.760
Yes	149 (14.8)	76 (15.2)	73 (14.5)	
No	856 (85.2)	425 (84.8)	428 (85.5)	
Hypertension				0.037*
Yes	164 (16.3)	94 (18.8)	70 (13.9)	
No	841 (83.7)	407 (81.2)	434 (86.1)	
Sleep disturbance				<0.001*
Yes	218 (21.7)	79 (15.8)	139 (27.6)	
No	787 (78.3)	422 (84.2)	365 (72.4)	
Stress				0.186
Normal	942 (93.7)	477 (95.2)	465 (92.3)	
Mild	33 (3.3)	11 (2.2)	22 (4.4)	
Moderate and above	30 (3.0)	12 (2.6)	17 (3.4)	
Dyslipidaemia				0.830
Yes	156 (15.5)	79 (15.8)	77 (15.3)	
No	849 (84.5)	422 (84.2)	427 (84.7)	
Recreational noise exposure				0.772
Yes	97 (9.7)	47 (9.4)	53 (10.5)	
No	908 (90.3)	454 (90.6)	451 (89.5)	
Environmental noise exposure				0.195
Yes	172 (17.1)	78 (15.6)	94 (18.7)	
No	833 (82.9)	423 (84.4)	410 (81.3)	

Effect of noise exposure on the risk of hypertension

**Figure 1:**
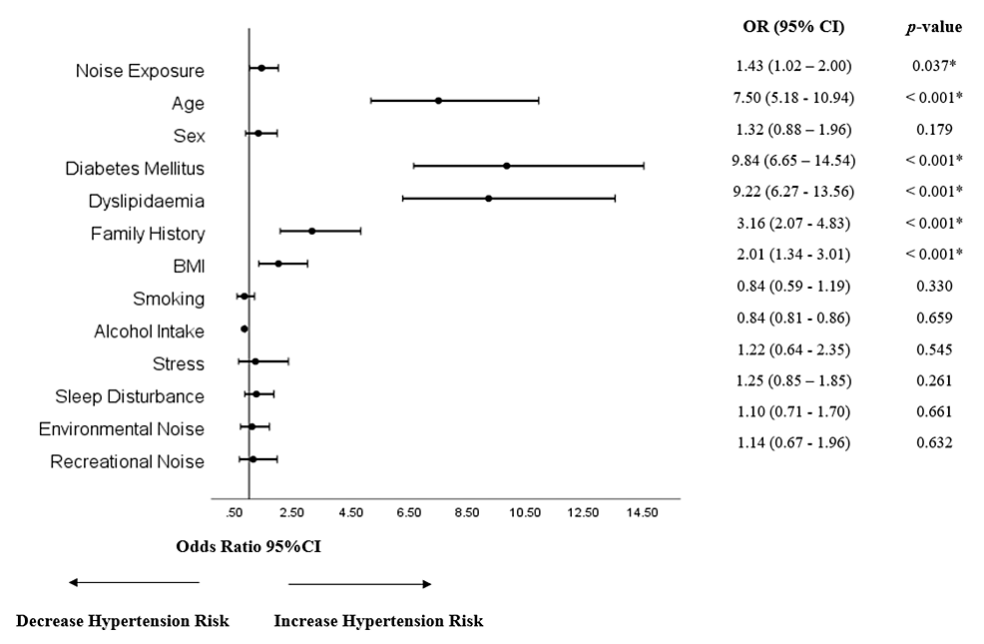
Univariate analysis of hypertension and its associated factors *p < 0.05 OR: odds ratio; CI: confidence interval

**Figure 2:**
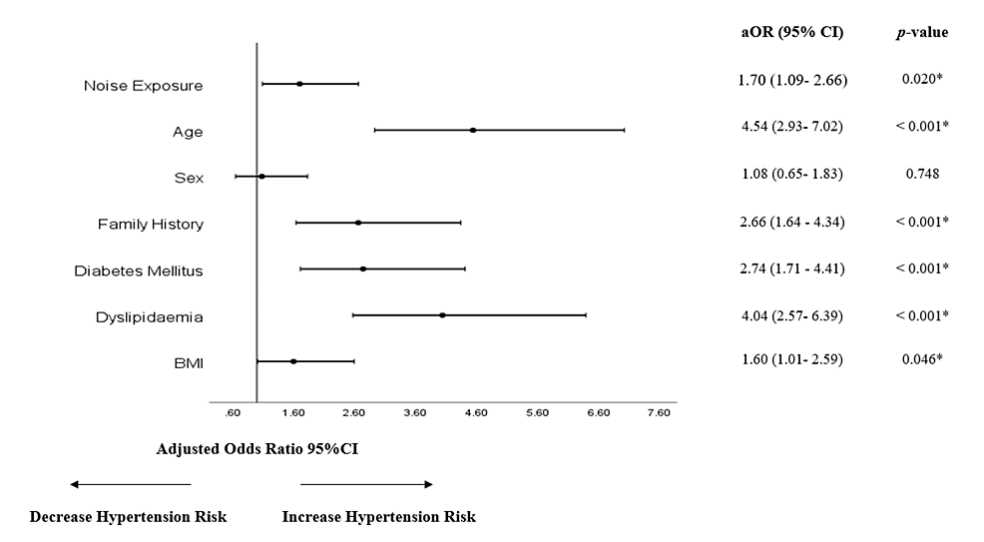
Multiple logistic regression of hypertension and its confounding factors *p < 0.05 Hosmer-Lemeshow test: p-value > 0.05 (0.098) aOR: adjusted odds ratio, adjusted for age, sex, family history, DM, dyslipidemia, BMI status; CI: confidence interval

Figure 1 shows the association of various risk factors with hypertension. The result showed that employees exposed to occupational noise had 43% higher odds of developing hypertension compared to those without exposure to noise exposure. This association was statistically significant, with a p-value of 0.037. After adjusting for confounders (age, sex, smoking, family history, diabetes, dyslipidaemia, BMI), the risk of hypertension was significantly higher in employees with noise exposure (OR 1.70, 95% CI 1.09-2.66) compared to non-noise-exposed employees as shown in Figure 2.

**Figure 3:**
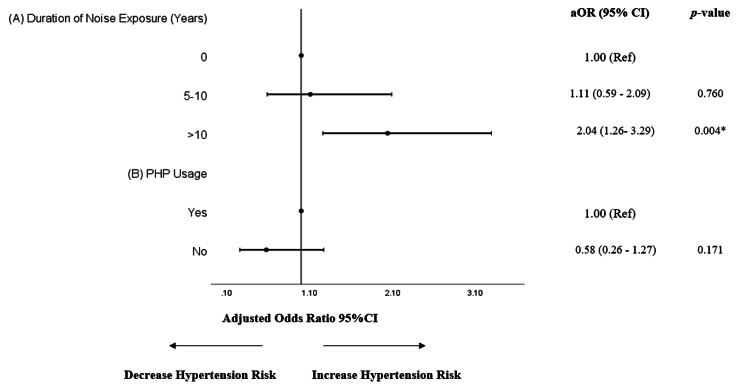
(A) Association between occupational noise exposure and hypertension according to duration of noise exposure (years). (B) Association between occupational noise exposure and hypertension according to PHP usage among noise-exposed employees Hosmer- Lemeshow Test: (A) p-value > 0.05 (0.116) and (B) p-value > 0.05 (0.682) *p < 0.05 aOR: adjusted odds ratio, adjusted for age, sex, family history, DM, dyslipidemia, BMI status; CI: confidence interval

Upon further analysis according to the duration of noise exposure, the risk of hypertension was heightened even further. The odds of developing hypertension for those with less than 10 years of noise exposure was 1.11 (95% CI 0.59-2.09), while for those with more than 10 years of exposure, the odds rose to 2.04 (95% CI 1.26-3.29). This trend was statistically significant only for the latter group, as shown in Figure 3 (A).

Upon further analysis according to PHP usage among noise-exposed employees, the risk of hypertension was higher among employees who wore PHP compared to those who did not, as shown in Figure 3 (B). However, this difference was not statistically significant.

## Discussion

This report has demonstrated that, both before and after accounting for potential confounding factors such as age, sex, DM, dyslipidemia, family history, and BMI, employees who were exposed to occupational noise are significantly more likely to develop hypertension compared to those without such exposure (p < 0.05). Additionally, employees with more than 10 years of noise exposure had an increased risk of hypertension, and the use of hearing protectors (self-reported) did not reduce the risk of hypertension among noise-exposed employees.

These findings align with previous research, indicating that individuals chronically exposed to continuous noise levels of at least 85 dB tend to have higher blood pressure compared to those without exposure. In a cross-sectional study of 22,906 U.S. civilians, a positive association was demonstrated between self-reported occupational noise exposure and hypertension [2]. Similar findings were also noted in a study of 1,559 steel workers in China. Self-reported exposure to loud workplace noise was found to be associated with hypertension and increased mean blood pressure (OR 2.03, 95% CI: 1.15-3.58), which is consistent with the findings presented in this report [19].

The study findings are in line with international studies indicating that the risk of hypertension gradually increases with a longer duration of occupational noise exposure. Two cross-sectional studies from China, one conducted among industrial workers in Nanjing and the other among steel factory workers in Guangzhou, found that workers with more than 10 years of occupational noise exposure were positively associated with hypertension [19,20]. A longer duration of noise exposure is generally considered more harmful and would be expected to have a greater impact on health outcomes. Therefore, it is not unexpected that these studies found a more significant association among those who worked in a noisy environment for a longer duration. These findings indicate the cumulative effect of noise exposure on hypertension in noise-exposed workers.

This study found that the use of hearing protectors (self-reported) did not reduce the risk of hypertension among noise-exposed employees. This is consistent with a previous study that analyzed the risk of hypertension according to the usage of PHP (self-reported) [21]. This may suggest that the effectiveness of PHP for noise protection is low, attributable to various factors. One such factor is the lack of knowledge about self-care among lower-educated workers. The absence of awareness about the importance of PHP can lead to poor health practices and risky behaviors, resulting in lower compliance with its use [22]. Additionally, discomfort due to heat and moisture buildup and communication interference may also reduce compliance rates [23]. Employees may also lack motivation to address workplace noise due to the gradual and uncertain nature of noise's effects on hypertension. The combination of these factors could contribute to PHP's decreased effectiveness, explaining the non-significant association found in this study. It was also observed that regular maintenance of noise protectors was not performed by the management team. Moreover, participants were noted to wear the required PHP only when supervisors were present, potentially biasing the data and suggesting a fear of being penalized for reporting non-compliance.

Hypertension, like many other NCDs, is caused by a combination of factors, each with a different level of association. In both models of this study, the relationship between occupational noise and hypertension was found to be weaker than some associations between other variables (such as age and diabetes) and hypertension. However, if the association between occupational noise and hypertension is indeed causal and noise-exposed workers are prevalent, then noise could still be an important contributor to the overall burden of hypertension. Despite the weaker association found, addressing noise exposure as a potential risk factor for hypertension remains important, especially in industries where it is prevalent.

This study is one of the few research projects that has focused on the association between occupational noise and hypertension in the ASEAN region, which has different cultural and lifestyle habits. For instance, in terms of dietary choices, East Asian has a higher average sodium intake of 4.8 g/day as compared to the global average sodium intake of 3.95 g/day [24]. In terms of cultural norms and beliefs, traditional and complementary medicine (TCM) is popular in many developing countries, including this region as it perceived that TCM has lower rate of side effects than Western medicine and TCM focus on quality of life when a cure is not possible [25]. These differences may influence the relationship between occupational noise and health outcomes. Another strength of the study is that it addresses an understudied and neglected topic in the ASEAN region and Malaysia. Moreover, the rigorous standards used to select participants for the study improved the internal validity of the study by reducing the risk of confounding due to other extraneous variables. The exclusion criterion of noise exposure less than five years, which was stricter than in other studies that may have used a criterion of noise exposure less than one year, helped to ensure that the study sample was more homogeneous and that the results were not influenced by noise exposure as it was too short of a duration to have an impact. Studies have found that the association between the duration of noise exposure and hypertension began to increase after a duration of more than five years and escalates rapidly after 10 years [20-21]. Additionally, limiting the sample to participants with no prior noise exposure allowed for better control of the effects of previous noise exposure on the study outcomes. As chronic diseases such as hypertension tend to develop gradually over time, the study population is appropriate given that almost 80% of the participants have been in the workforce for more than 10 years, and almost two-thirds of the noise-exposed population have been exposed to noise for a significant period of time. This suggests that the study population has likely been exposed to noise for a sufficient duration to potentially impact their health outcomes.

One major limitation of the study is its inability to determine the incidence of hypertension among noise-exposed workers. A longitudinal study might offer the ability to ascertain this [26]. In addition, the noise exposure status was self-reported in this study, which can lead to biased estimates; however, previous research has shown that self-reported estimates of noise levels can be reliable and generally consistent with objective measures of noise levels [27-29]. Participants' responses were obtained through self-reported questionnaires, which could be susceptible to information and recall bias. This susceptibility arises from uncertainties about the accuracy of the information provided by participants, influenced by factors such as memory or perception. Another potential limitation is the presence of the healthy worker effect. This may be particularly relevant, as hypertension typically develops gradually over time, and the condition may manifest after workers have left the noisy job. This implies that the study population, consisting only of currently employed workers, may not accurately represent the overall health status of the exposed population. These limitations suggest that the true adverse effects of occupational noise on the worker population may be greater than what has been observed in this study. Multivariate analysis was conducted to control for potential confounders such as age, reducing the effect of this bias. Lastly, physical activity and dietary intake were not taken into consideration in this study. Physical activity is closely associated with hypertension; however, it was not included in further models as it is highly correlated with BMI. BMI is an acceptable measurement for determining the physical activity level in a given population [30].

## Conclusions

This study suggests that occupational noise hazards can significantly increase the risk of hypertension, particularly with prolonged exposure. These findings highlight the substantial impact of occupational noise hazards on hypertension and underscore the importance of addressing this issue to protect the health of employees. The results of this study are crucial for the basis of further research on this subject, which can be used for workplace interventions and changes in legislation regarding noise exposure, taking into account effects on the non-auditory system. The additional information obtained from this study is invaluable for understanding the true impact of noise exposure and for developing effective strategies to mitigate its harmful effects. By tailoring these strategies to specific occupations, a targeted approach can be adopted in public health to protect the well-being of employees.
